# Color-filter-array-based multispectral photoplethysmography optical sensor and its motion artifact correction algorithm

**DOI:** 10.1117/1.JBO.31.3.037001

**Published:** 2026-03-16

**Authors:** Yunfen Wei, Shu Cong, Runchao Yan, Zekai He, Hong Li, Gongming Yu, Mei Zou

**Affiliations:** a Kunming University, School of Physical Science and Technology, Kunming, China; b Nanjing University, National Laboratory of Solid State Microstructures, Nanjing, China

**Keywords:** multi-stage least mean square adaptive filter, motion artifact, blind source separation, photoplethysmography, color filter, physiological parameter estimation

## Abstract

**Significance:**

The performance of wearable biosensors is highly influenced by motion artifacts (MAs).

**Aim:**

We propose a motion artifact removal algorithm using blind source separation–multi-stage least mean square adaptive filtering with multi-wavelength photoplethysmography (PPG) signals to enable accurate physiological parameter estimation in wearable devices.

**Approach:**

The algorithm is implemented with a custom-designed PPG sensor that enables synchronized multi-wavelength acquisition via a compact optical design integrated with a color filter array. The algorithm exploits the high correlation of MA components across wavelengths to autonomously generate a noise reference in real time through blind source separation. Furthermore, a frame-level quality assessment mechanism based on power spectral entropy is introduced, which dynamically evaluates the interference level according to the entropy value and intelligently switches between two pre-optimized sets of filter parameters. This allows for dynamic parameter adjustment of the MSLMS filter, thereby effectively tracking and suppressing motion artifacts without the need for external inertial sensors.

**Results:**

The performance of the proposed algorithm was evaluated in a study involving 13 subjects performing free-arm swings to simulate daily motion. Experimental results demonstrate that after algorithm processing, the limits of agreement between the estimated heart rate and the electrocardiogram reference values narrowed from [3.09, 26.94] to [−1.98,2.11]  bpm, the Pearson correlation coefficient improved from 0.86 to 0.99, and the mean absolute error significantly decreased from 15.12 to 0.76 bpm.

**Conclusions:**

We present an integrated hardware–algorithm co-design, offering a practical solution for high-precision and robust physiological monitoring in ambulatory settings using wearable devices.

## 1 Introduction

This photoplethysmography (PPG) is a noninvasive optical technique for monitoring blood volume changes at the skin surface.^1^ PPG signals reflect blood flow dynamics, enabling continuous measurement and monitoring of physiological parameters such as heart rate (HR), blood oxygen saturation, and blood pressure.^2^ Owing to its noninvasive design, simplicity, and low cost,^3^ PPG sensors are widely used in various vital sign monitoring applications.

Despite its advantages, PPG still faces several challenges in both daily activities and clinical environments. Although it generally provides accurate measurements in the stationary state, its performance degrades significantly during motion due to various sources of noise, including power line interference, ambient light (AMB), and motion artifacts (MAs).^4^ Among these, motion artifacts are the most significant factor affecting PPG signal quality. They are primarily caused by relative movement between the sensor and the skin, which alters the angle of light incidence and optical path, as well as by deformation of subcutaneous tissue structures.^5^ Power line interference can be effectively mitigated using filtering techniques.^6^ Ambient light interference is commonly addressed through methods such as correlated double sampling (CDS) or by applying optical filters to photodiodes (PDs).^7^ CDS operates by calculating the difference between photodetector signals at two time points, effectively eliminating ambient light interference. Optical filters, on the other hand, physically block unwanted light wavelengths, thereby improving signal clarity at the hardware level.

However, the high sensitivity of PPG signals to motion artifacts remains a critical technical barrier. Even minor patient movements—such as hand tremors, postural shifts, or coughing—can cause substantial waveform distortions, compromising the accuracy and clinical reliability of key physiological parameters such as HR and oxygen saturation. This limitation significantly hinders the clinical application of PPG in dynamic conditions, including intraoperative monitoring, daily activity tracking, and large-scale deployment in wearable devices. Consequently, researchers are actively seeking solutions at both the hardware and algorithmic levels to enhance the robustness of PPG signals in complex clinical settings and to improve their clinical usability and reliability.

Numerous methods have been proposed to mitigate motion artifacts, including wavelet denoising,^8^ independent component analysis (ICA),^9^ empirical mode decomposition (EMD),^10^ and adaptive filtering.^11^ Although these methods can effectively reduce artifacts in dynamic conditions and improve PPG signal fidelity to some extent, they struggle to remove artifacts when the frequency of motion artifact overlaps with the frequency of the PPG signal. Adaptive filtering can remove unwanted components regardless of their frequency components.^12^ However, adaptive filtering algorithms typically rely on additional motion sensors such as an accelerometer or a gyroscope to generate noise reference signals.^13^^–^^15^ Mohan et al.^16^ proposed an accelerometer-based adaptive noise cancellation technique to reduce errors in arterial oxygen saturation measurements. However, the recorded acceleration frequently fails to accurately reflect true motion. To address this limitation, Lee et al.^17^ replaced the accelerometer with a gyroscope for noise signal acquisition. Their results demonstrated that the gyroscope-assisted approach outperformed the accelerometer-based method, particularly during walking. Nonetheless, this approach remains limited because the motion sensors do not accurately represent the motion artifacts in PPG signals, resulting in low correlation between measured artifacts and actual PPG disturbances, and the additional hardware increases both system size and cost.

To address this challenge, researchers have developed non-motion sensor approaches that derive noise reference signals directly from PPG recordings. These methods utilize singular value decomposition,^18^ ICA,^19^ and EMD^20^ to extract noise reference signals from contaminated PPG recordings and thereby suppress motion artifacts. Zhang et al.^21^ introduced an adaptive filtering framework that benchmarks noise references produced by EMD, ensemble EMD (EEMD), and complete EEMD with adaptive noise (CEEMDAN). They demonstrated that CEEMDAN offers superior noise separation performance in scenarios where PPG and motion artifact spectra overlap. However, these approaches have two significant limitations: (1) they require multiple complex stages—signal decomposition, mode selection, and reconstruction—leading to high computational complexity, and (2) the generated noise references are not physically correlated with real motion sources, compromising the physiological basis of the noise model.

Recently, adaptive filtering techniques leveraging multi-wavelength PPG signals for motion artifact removal have been developed. Yousefi et al.^22^ proposed a two-stage normalized least mean square (NLMS) adaptive algorithm, which effectively suppressed artifacts caused by tissue deformation and venous pulsation. Building on this, Lee et al.’s team^23^ developed a multi-channel PPG measurement system in 2020, integrating ICA for HR estimation in high-motion conditions (e.g., running or fast walking). Park et al.^24^ further advanced this direction by leveraging the strong correlation of MA components in closely spaced red/green light-emitting diode (LED) signals with distinct alternating current (AC) and direct current (DC) ratios and employed a subspace LMS (SS-LMS) filter for artifact removal without motion sensors. However, these methods rely on expensive signal processors and assume high inter-wavelength correlation, which in practice depends heavily on precise spatiotemporal synchronization, making sensor deployment challenging.

In this paper, we present a hardware–algorithm co-designed system for robust, motion-robust physiological monitoring. At its core is a novel, low-cost multi-wavelength PPG sensor that employs a color filter array integrated directly onto a photodiode array. This design enables truly synchronous acquisition of red (660 nm) and infrared (940 nm) signals through physical wavelength separation, eliminating the need for complex sequential driving or expensive processors. Crucially, this hardware ensures that MA exhibit inherently high spatiotemporal correlation across wavelengths—a physical prerequisite we experimentally verify.

Leveraging this correlated MA foundation, we propose a frame-selective blind source separation–multi-stage least mean square (BSS-MSLMS) adaptive filtering algorithm. The algorithm first uses blind source separation (BSS) to dynamically construct a high-fidelity noise reference from the multi-wavelength signals, eliminating dependency on external motion sensors. Its key innovation is an entropy-based, state-aware decision mechanism that assesses signal contamination in real time and intelligently switches between pre-optimized filter parameter sets. This design completely bypasses the “static convergence period” required by conventional adaptive filters, enabling immediate response to sudden motion and robust suppression of spectrally overlapping artifacts.

Experimental results validate the synergy of this co-design: the Pearson correlation between estimated and reference heart rate reached 0.99, with a mean absolute error of only 0.76 bpm. By integrating a compact, low-cost hardware front-end with an adaptive, convergence-free algorithm, this work provides a practical and scalable “high-accuracy, low-cost, and compact” solution for wearable health monitoring in dynamic environments.

The remainder of this paper is organized as follows. Section 2 reviews the optical principles of PPG sensors, the design of a novel PPG module, and optical simulations to optimize LED-PD spacing. Section 3 quantifies the different noise components from various signal sources, characterizes motion artifacts, and discusses optimal noise reference generation in adaptive filtering. Section 4 presents the experimental setup. Section 5 reports the measurement results. Section 6 concludes the paper.

## 2 Design of the Novel Multi-Wavelength PPG Sensor Using Color Filter Array

### 2.1 Working Principle

**Fig. 1:**
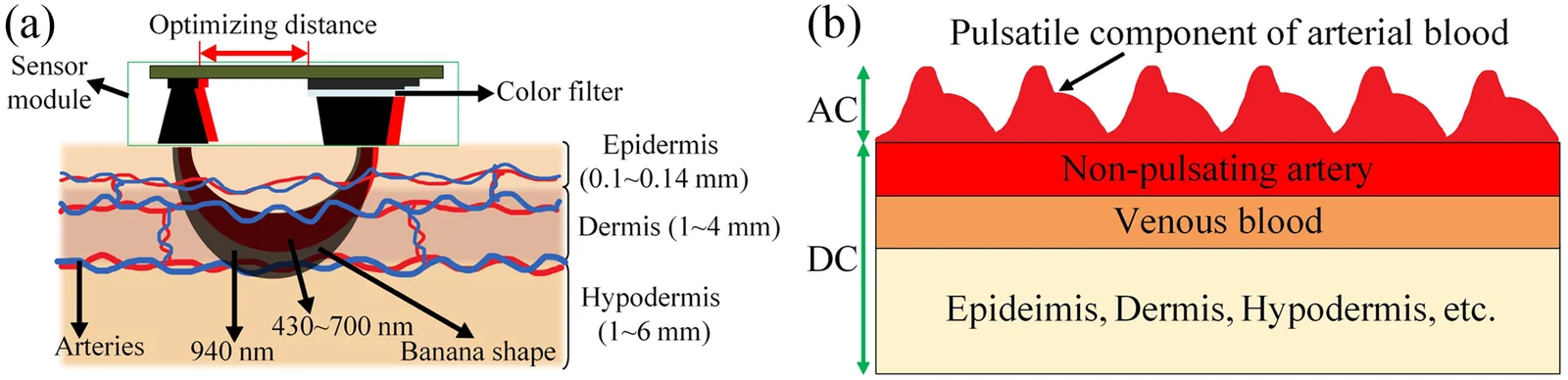
(a) Schematic of a reflective PPG sensor in the dermal vascular bed and the banana-shaped light propagation model. (b) PPG signal components.

PPG is an optical method for measuring blood volume changes by shining light on the skin using an LED and detecting the reflected or transmitted light using a PD. The LED light penetrates the skin and is partially attenuated due to absorption by biological tissues. Wrist-worn PPG sensors commonly use a reflective detection mode, as illustrated in Fig. 1(a). The light travels from the LED to the PD through highly scattering layers, including the epidermis, dermis, hypodermis, and pulsating capillaries and arterioles. According to the Beer–Lambert law, most light reaching the PD follows a banana-shaped path, and longer wavelengths penetrate more deeply into tissue.^25^ The light attenuation caused by scattering and absorption in tissues, blood, and vessel walls can be characterized by changes in the absorption coefficient,^3^ i.e.

$$A_{p\lambda }=-\log\;\frac{L_{TP\lambda }}{L_{IP\lambda }}=\mu_{a\lambda }.l_{P\lambda },$$ (1)

where the subscript p denotes the specific optical path within the banana-shaped propagation trajectory, λ represents the wavelength of the light path, $\mu_{a\lambda }$ corresponds to the absorption coefficient along the defined optical path, $l_{P\lambda }$ indicates the emission power of the LED under the specified path, $L_{TP\lambda }$ is the light intensity of the transmitted light in the light trace in the banana shape, $L_{IP\lambda }$ is the light intensity of the incident light along the banana, and $A_{P\lambda }$ defines the total optical absorption from the LED to the PD.

As illustrated in Fig. 1(b), the PPG signal contains AC and DC. The AC component reflects the rhythmic changes in blood volume caused by heartbeats,^26^ whereas the DC component captures the baseline signal and includes low-frequency variations related to respiration, autonomic nervous activity, and body temperature regulation.^27^ In reflective PPG sensors, the distance between the LED and PD plays a critical role in performance. If the distance between the LED and PD fails to correspond to the optimal “banana size” for a given wavelength, it may result in the PD being unable to capture the light emitted by the LED and reflected back. Alternatively, the light power received by the PD may be insufficient, thereby compromising the measurement accuracy and the overall signal quality of the sensor.^28^ Moreover, the pulsating signal detected by the PD is often affected by noise from ambient light, motion artifacts, and interference related to photodetectors. Therefore, to achieve robust multi-wavelength physiological parameter monitoring, it is essential to effectively manage ambient light and motion interference at the hardware level. Accordingly, this subsection presents the design and implementation of a novel multi-wavelength synchronous acquisition PPG sensor based on a color filter array. The design utilizes a broadband LED light source. By integrating wavelength-specific color filters above the PD array, it enables synchronous separation and acquisition of PPG signals across different spectral bands. To optimize performance, the optimal LED-PD spacing is determined through optical simulation for each wavelength.

### 2.2 Optical Simulation

**Fig. 2:**
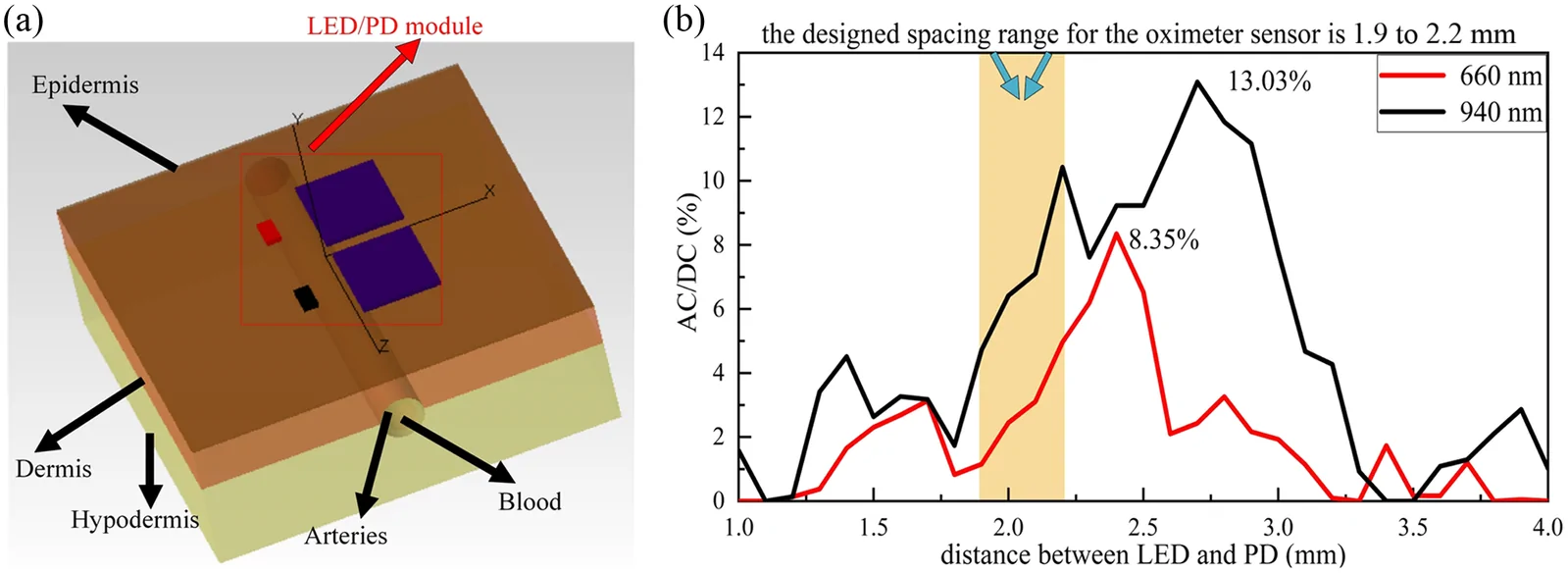
(a) Optical simulation model. (b) Power received by the PD, expressed as the AC/DC ratio for maximum signal-to-noise ratio, under varying LED distances and wavelengths.

Optical simulations were conducted using TracePro to construct the model shown in Fig. 2(a), which comprises the LED-PD module, epidermis, dermis, hypodermis, radial artery, and blood. In the LED-PD module, the PD dimensions are 5  mm×4.24  mm, and the LED dimensions are 1.6  mm×0.8  mm. The tissue layer thicknesses are set as follows: epidermis (0.1 to 0.14 mm), dermis (1 to 4 mm), and hypodermis (1 to 6 mm).^29^ Table 1 summarizes the optical properties used in the simulation, including thickness, refractive index, absorption, scattering coefficient, and anisotropy, to enable accurate modeling. The radial artery pulses at a depth of 2 to 2.4 mm. The LED-PD spacing was varied from 1 to 4 mm in 0.1-mm increments to simulate and identify the distance that maximizes the AC/DC ratio of the PPG waveform at the PD. The results are shown in Fig. 2(b). The curves exhibit similar overall trends but with numerical deviations, primarily attributable to differences in the propagation, absorption, and scattering properties of the wavelengths within tissue. The 660-nm wavelength has higher tissue absorption and scattering coefficients, resulting in shallower penetration depth. It is more susceptible to superficial tissue structures and optical path perturbations, leading to significant signal attenuation with increasing distance. In contrast, the 940-nm wavelength has a lower absorption coefficient and deeper penetration, enabling the acquisition of more stable subcutaneous reflectance information. Consequently, it can maintain a higher signal-to-noise ratio and stable AC/DC output even at larger distances. Within the LED-PD distance range of 1.9 to 2.2 mm, the AC/DC ratios for both wavelengths exhibit a relatively stable and nearly linear increase, which facilitates reliable AC signal extraction. However, the AC/DC values become unstable when the distance is ≥3  mm. Therefore, the 1.9- to 2.2-mm range is identified as the optimal LED-PD distance for achieving synergistic multi-wavelength signal acquisition.

**Table 1:** Bio-optical properties of human tissues: reflectance and scattering parameters.

Materials	Thickness (mm)	Refractive index (n)	Absorption ($\mu_{a}$) (1/mm)	Scattering anisotropy (g)	Scattering coefficient ($\mu_{s}$) (1/mm)
Epidermis	0.14	1.4	0.23 @ 660 nm	0.8	20.00 @ 660 nm
			0.06 @ 940 nm		16.00 @ 940 nm
Dermis	2.4	1.5	0.14 @ 660 nm	0.8	14.00 @ 660 nm
			0.08 @ 940 nm		9.700 @ 940 nm
Hypodermis	5	1.44	0.36 @ 660 nm	0.8	11.90 @ 660 nm
			0.08 @ 940 nm		8.900 @ 940 nm
Arteries	2 (DC), 2.4 (AC)	1.4	1.70 @ 660 nm	0.94 @ 660 nm	732.0 @ 660 nm
			5.96 @ 940 nm	0.96 @ 940 nm	700.0 @ 940 nm
Blood	1 (DC), 1.6 (AC)	1.36	2.30 @ 660 nm	0.93 @ 660 nm	989.0 @ 660 nm
			6.60 @ 940 nm	0.96 @ 940 nm	800.0 @ 940 nm

### 2.3 A Novel Multi-Wavelength PPG Sensor with Color Filter Array

**Fig. 3:**
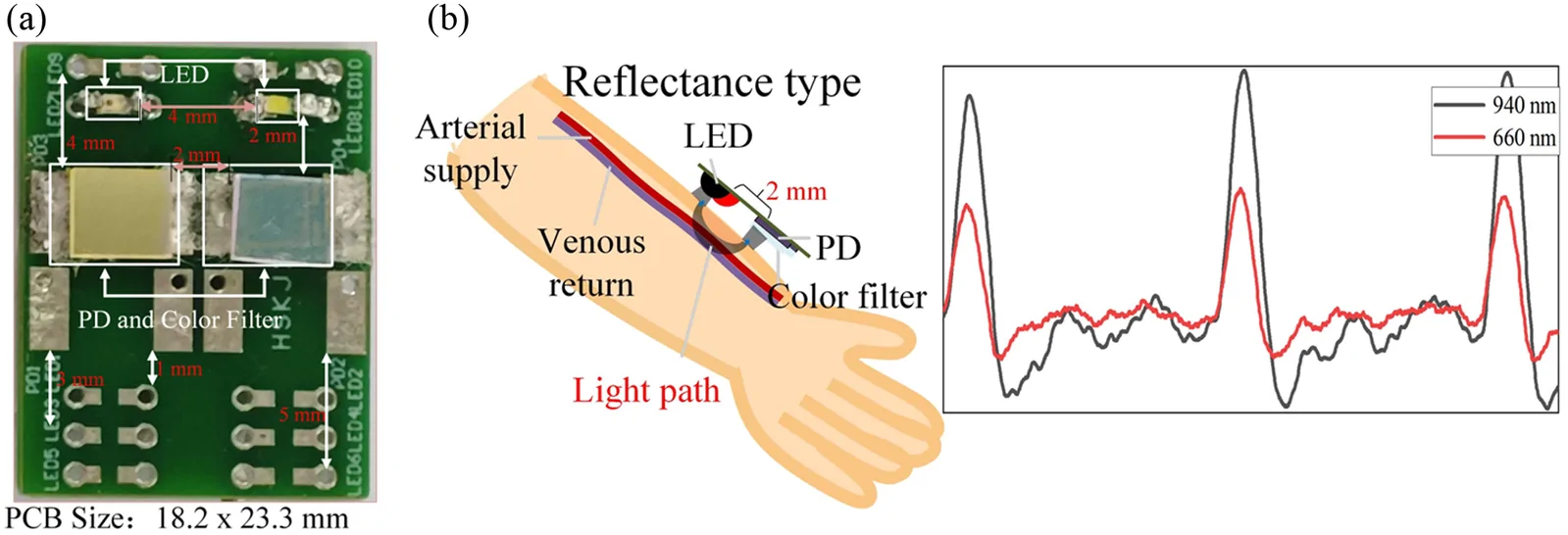
(a) Synchronous multi-channel PPG sensor module. (b) Multi-wavelength PPG signals measured at an LED-PD distance of 2 mm using the wrist-worn reflective PPG sensor with color filters.

Based on the aforementioned simulation results, a compact multi-channel PPG synchronous acquisition sensor module was designed using Altium Designer and fabricated. A photograph of the fabricated sensor is shown in Fig. 3(a). The color filter array is directly attached to the surface of the PD array using a thin layer of optical adhesive. This configuration ensures precise wavelength selection and effective ambient light rejection for each channel. This hard-coated dielectric band-pass filter array comprises two independent units with center wavelengths (CWLs) precisely aligned to 660 nm (red) and 940 nm (infrared). Its operation relies on wavelength-selective transmission: each unit efficiently transmits only its targeted narrow band (around the CWL) while strongly attenuating out-of-band light, including emissions from non-target LEDs and ambient stray light.

**Fig. 4:**
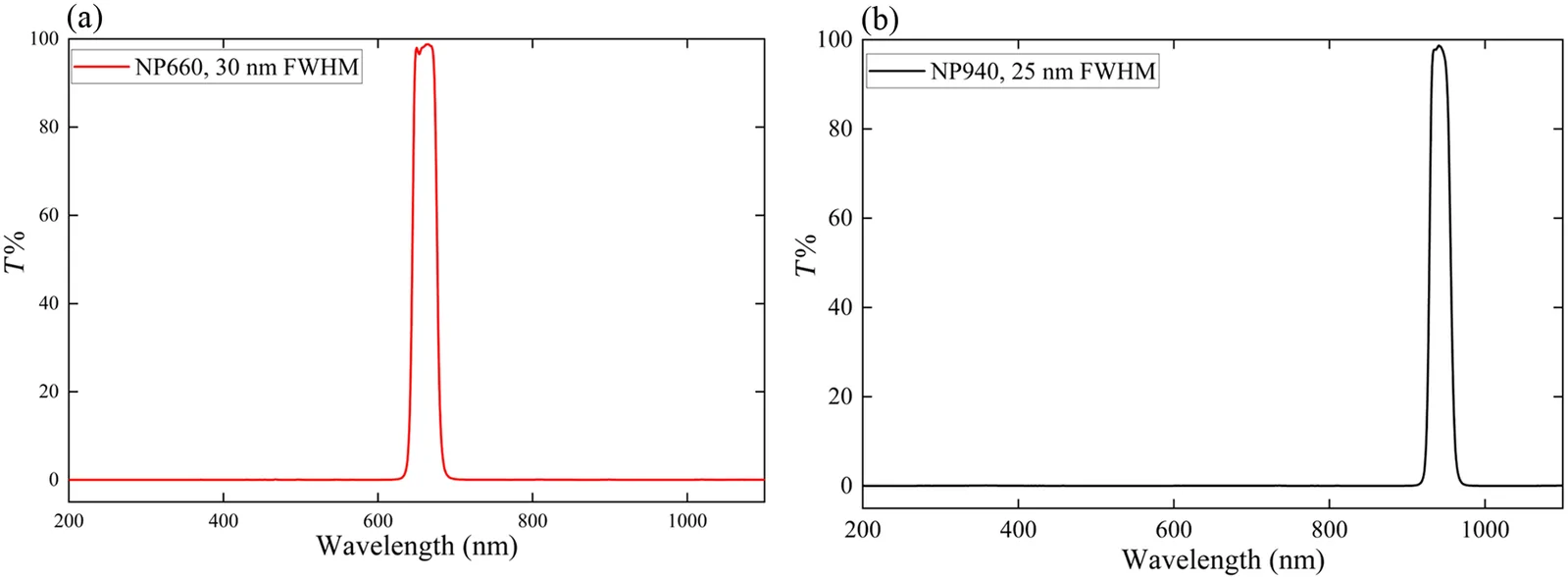
Measured spectral transmittance curves of the multi-channel filters integrated into the sensor. (a) 660-nm red-light channel filter. (b) 940-nm infrared-light channel filter. Data provided by the manufacturer.

The key optical performance parameters of both filters were quantified using the measured spectral transmittance curves provided by the manufacturer [Figs. 4(a) and 4(b)], exhibiting narrow band-pass characteristics at their target CWLs. Analysis of these curves yielded the core characteristics summarized in Table 2. Both filters demonstrate a peak transmittance exceeding 98% at their CWL, with narrow bandwidths [full width at half maximum (FWHM) of ∼30 and ∼25  nm, respectively] and strong out-of-band rejection (transmittance typically <0.1%). These properties ensure that each PD channel receives a pure target-wavelength PPG signal while minimizing ambient light interference and optical crosstalk. The sensor employs a white LED (430 to 700 nm, 0603HW19-D) and an infrared LED (940 nm, KP-608F3C) as light sources. The PDs (TEMD5080X01) exhibit a spectral response range of 350 to 1100 nm, ensuring effective sensitivity across the target bands. To experimentally validate the influence of LED-PD spacing on signal quality, measurements were performed over a spacing range from 1 to 5 mm in 1-mm increments. As shown in Fig. 3(b), at a spacing of 2 mm, both channels acquire PPG waveforms with a high signal-to-noise ratio and distinct morphological features. Based on the integration of simulation and experimental results, the optimal LED-PD spacing was determined to be 2 mm.

**Table 2:** Key optical parameters of the integrated color filter array.

Parameters	Center wavelength (nm)	FWHM (nm)	Peak transmittance (%)	Typical out-of-band transmittance (%)
660-nm channel (red)	660	∼30	∼98	<0.1
940-nm channel (infrared)	940	∼25	∼98	<0.1

Compared with traditional PPG sensors, this design—featuring multi-wavelength LED synchronous driving and PD-integrated color filters—fundamentally addresses inherent limitations of conventional approaches, such as inter-signal phase errors from sequential switching and ambient light interference due to insufficient optical filtering. Moreover, the customizable nature of the filter array affords flexible wavelength selection and scalability, providing a more reliable hardware foundation for multi-wavelength-based physiological parameter estimation algorithms.

### 2.4 Verification of Multi-Channel Signal Characteristics and Motion Artifact Correlation

**Fig. 5:**
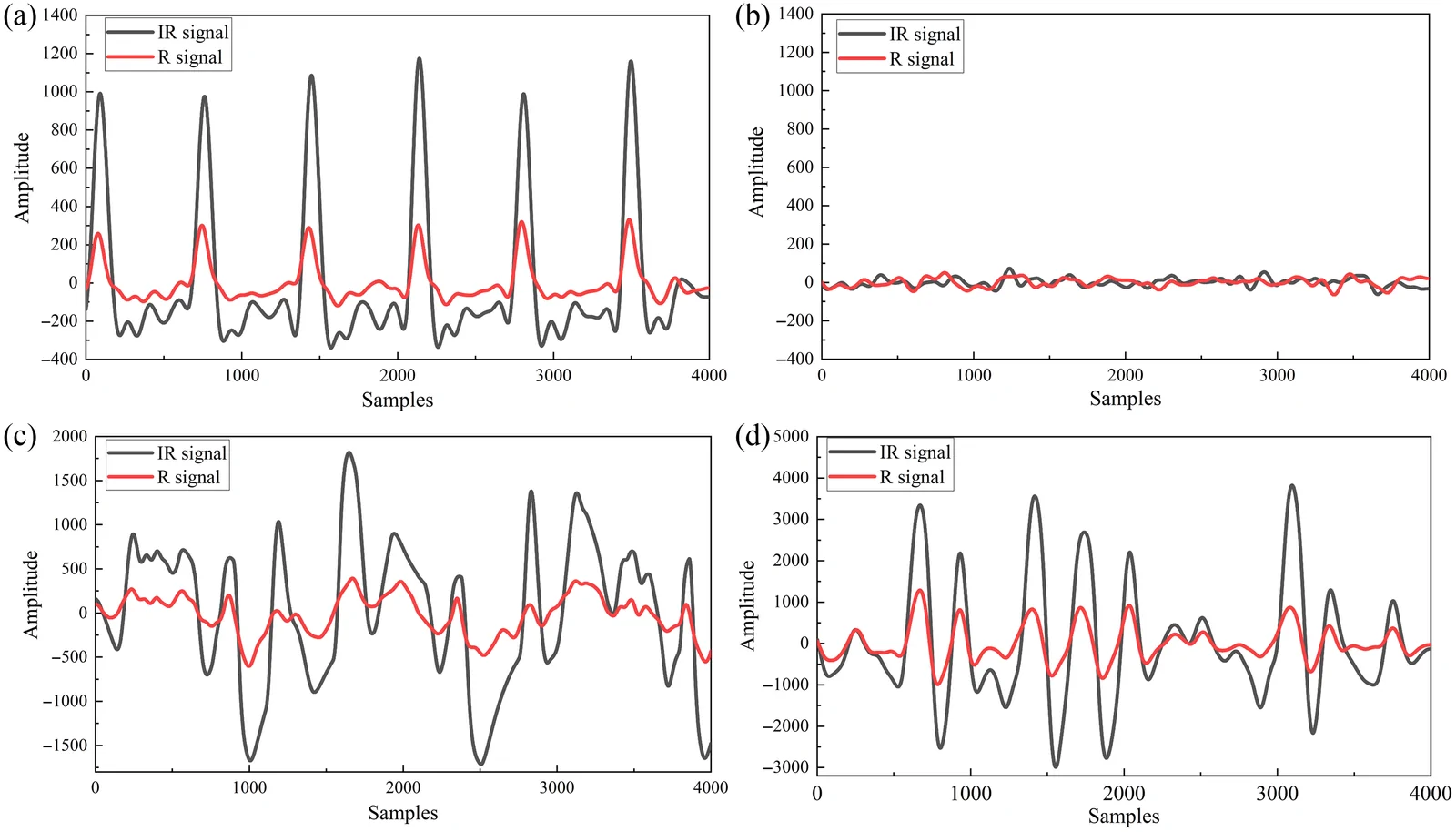
Experimental results of the blood flow occlusion test. (a) Multi-wavelength PPG signals in the stationary state before occlusion (0.5 to 5 Hz filtered). (b) Multi-wavelength PPG signals in the stationary state after occlusion [processed identically to panel (a)], showing suppression of the AC component. (c) Pure motion artifacts from multi-wavelengths: extracted via 0.5- to 15-Hz bandpass filtering and normalization from signals recorded during arm swinging under occlusion. (d) Multi-wavelength PPG signals with motion artifacts: acquired during arm swinging after occlusion release [processed identically to panel (a)], representing a superposition of the AC and MA components.

Based on the hardware described in Secs. 2 and 3, this section analyzes the characteristics of the synchronously acquired 660- and 940-nm PPG signals. The multi-channel system exhibits three progressively supportive features:

First, as shown in Fig. 5(a), despite different tissue absorption, the AC components of both wavelengths originate from the same arterial pulsation. In the stationary state, their average correlation reaches 0.94, confirming physiological homogeneity and enabling joint signal processing.

Second, as shown in Fig. 5, the optimized hardware—including a 2-mm LED-PD spacing, synchronous driving, and a precision filter array—delivers high-quality, temporally aligned raw signals with excellent SNR under both static and motion conditions.

**Fig. 6:**
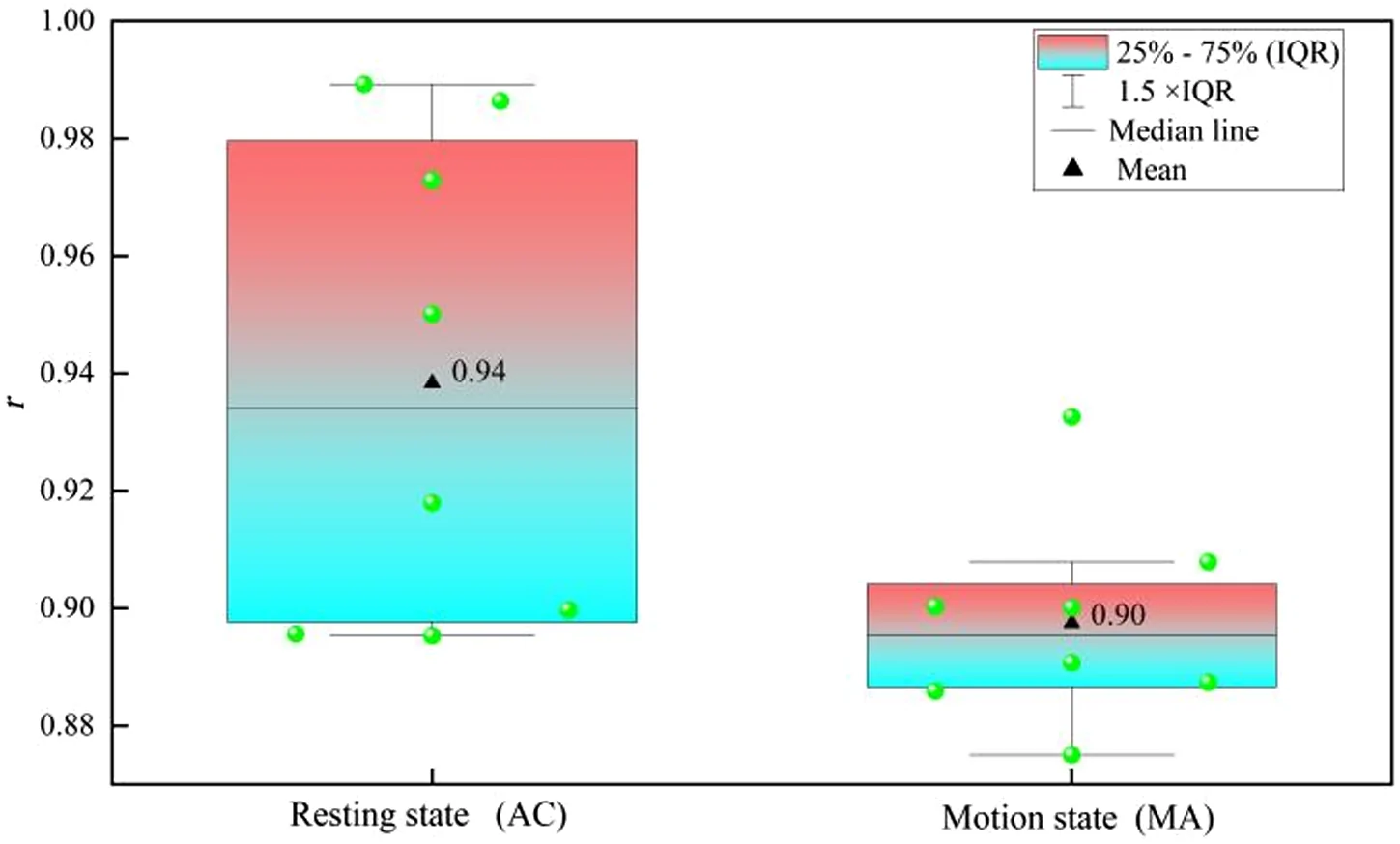
Box plot comparison of the inter-channel Pearson correlation coefficient (r) between conditions dominated by motion artifacts and stationary physiological signal conditions.

Third, and most critically, MA shows high cross-channel correlation. Because the two channels share the same packaging, optical path, and skin interface, physical perturbations modulate both signals almost identically. This was verified through an upper-arm blood flow occlusion test, which suppressed the pulse so that the recorded signals during arm swinging primarily contained MA. As shown in Fig. 5(c), the MA waveforms exhibit clear synchrony. Population analysis (Fig. 6) further shows that under motion-dominant conditions, the median inter-channel correlation is ∼0.90—slightly lower than the stationary-state physiological correlation (∼0.93) but still within the strong correlation range. This confirms that motion-induced artifacts possess strong spatiotemporal consistency across wavelengths.

In summary, the multi-channel signals provide physiological coherence, high signal quality, and highly correlated motion artifacts. The strong MA correlation forms the key link between the hardware design and the subsequent algorithm, enabling motion-artifact modeling and cancellation without external sensors, solely through the intrinsic relationship between the two wavelengths.

## 3 Motion Artifact Cancellation

Adaptive filters can eliminate noise signals regardless of their frequency components. The MA algorithm developed based on adaptive filters requires the reference signal to be strongly correlated with the artifact signal but uncorrelated with the pulse signal.^30^ The designed synchronous multi-wavelength PPG sensor provides the crucial hardware foundation for MA suppression without external motion sensors, as the motion artifact components in the signals acquired from its red and infrared channels exhibit high correlation due to their origin from common physical perturbations. This paper presents a motion artifact removal algorithm based on the BSS-MSLMS adaptive algorithm, where BSS is a simple batch learning algorithm for semi-blind extraction of a desired source signal with temporal structure from linear mixtures.^31^ The (MSLMS) adaptive filter employs a hierarchical adjustment of parameters (mu,N), dynamically optimizing weights through instantaneous gradient estimation to minimize the output error.

**Fig. 7:**
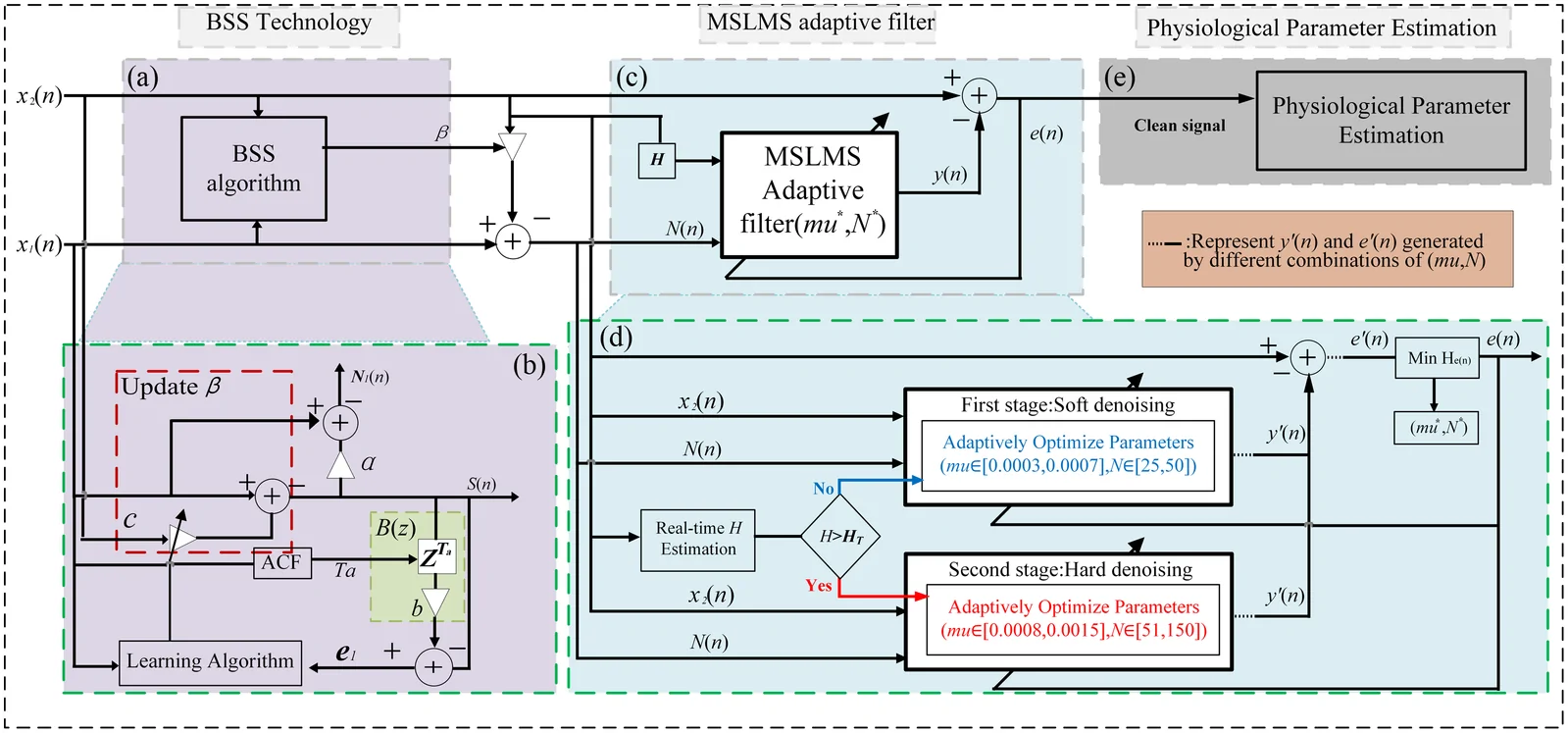
(a)–(e) BSS-MSLMS adaptive algorithm framework diagram.

As shown in Fig. 7, the algorithm first leverages the strong MA correlation among the multi-wavelength signals. It employs BSS to estimate a parameter β online, thereby constructing a noise reference signal highly correlated with the MA in the infrared channel. Subsequently, a frame-level quality assessment mechanism based on power spectral entropy is introduced. This mechanism dynamically evaluates the signal contamination level and intelligently switches between two pre-optimized filter parameter sets accordingly—employing a small learning rate and low filter order under low-entropy conditions to preserve signal details and fidelity and switching to a large learning rate and high order under high-entropy conditions to rapidly track and suppress intense motion artifacts. Furthermore, parameters are fine-tuned within each frame via grid search, aiming for locally optimal filtering by minimizing the error entropy. Through the collaborative mechanism of “BSS-based reference generation – entropy-guided strategy selection – MSLMS adaptive filtering – real-time parameter optimization,” the algorithm achieves adaptive and robust tracking suppression of motion artifacts. This effectively overcomes the limitations of traditional adaptive filters, which rely on fixed parameters and a static convergence period, thereby providing a reliable solution for high-accuracy heart rate monitoring based on PPG in dynamic environments.

### 3.1 Modeling and Representation of Motion Artifacts

There is minimal correlation between the useful periodic pulsating component and motion artifacts in the PPG signal. Therefore, the components are assumed to be statistically independent. When motion artifacts and ambient light are introduced, the PPG signal can be expressed as

PPG=AC+DC+MA+AMB, (2)

where AC is the pulsating component required for extracting physiological parameters, DC is the artifact-free DC component, MA is the motion artifact, and AMB is the ambient light component. The DC component is effectively attenuated through high-pass filtering, and the AMB component, induced by ambient light, can be eliminated using a color filter.

By leveraging the synchronized multi-wavelength acquisition capability of the proposed PPG sensor, the MA components in signals of different wavelengths exhibit a high degree of correlation, as they originate from the same physical motion. When PPG signals are synchronously acquired using infrared and red light LEDs, with both the DC and AMB components effectively suppressed, the linear equations for the multi-wavelength PPG signals can be expressed as

$$\text{Red light signal}:\;x_{1}={\mathrm{AC}}_{R}+{\mathrm{MA}}_{R}=a{\mathrm{AC}}_{\mathrm{IR}}+c{\mathrm{MA}}_{\mathrm{IR}},$$ (3)

$$\text{Infrared light signal}:\;x_{2}={\mathrm{AC}}_{\mathrm{IR}}+{\mathrm{MA}}_{\mathrm{IR}},$$ (4)

where a = ACR/ACIR, c = MAR/MAIR, with subscripts R and IR denote red light and infrared light.

A noise reference signal N(n), related to both infrared and red light, is constructed using parameters β to eliminate motion artifacts in the infrared light signal. The noise reference signal is

$$N(n)=x_{1}-\beta x_{2}=(a-\beta ){\mathrm{AC}}_{\mathrm{IR}}+(c-\beta ){\mathrm{MA}}_{\mathrm{IR}}.$$ (5)

In this equation, AC represents the periodic component with temporal structure, and the correlated signal contains the AC component (β=c), the MA component (β=a), or a combination of these two sources. Therefore, by optimizing the parameters β, a reference signal containing only the motion artifact component can be generated.

### 3.2 Computation of Parameter ***β*** Using Blind Source Separation Technique

To optimize parameter β, a BSS model is constructed as shown in Figs. 7(a) and 7(b), weighted subtracter, predictor filter, and a simple FIR filter. This model represents a robust batch algorithm based on the BSS technique for continuously extracting noisy biomedical signals.

This algorithm first extracts a scaled estimate of the AC component in $x_{2}$ by minimizing the variance of the error signal $e_{1}(n)$. The error signal $e_{1}(n)$ can be mathematically expressed as

$$e_{1}(n)=S(n)-bS(n-T_{a}),$$ (6)

where $S(n)=x_{1}-c$ $x_{2}$, and b is the vector of coefficients of a simple FIR filter with a single delay of $Z^{-Ta}$, where $T_{a}$ is the period of the arterial source signal in discrete time, extracted using the autocorrelation function.^32^

Optimization of c and coefficient of b can be done by minimizing the mean square error defined as the cost function

$$J(c,b)=E[e_{1}^{2}(n)]=E[S^{2}(n)]-2bE[S(n)S(n-T_{a})]+b^{2}E[S^{2}(n-T_{a})],$$ (7)

where E[ ] denotes the expectation operator.

When the gradients of the cost function with respect to c and b are zero, the prediction error has its minimum value. So, by equating the gradient of the cost function with respect to c and b to zero, a system of equations can be derived. Solving these equations yields the update rules for c and b

$$c=\frac{-E[x_{1}^{2}]E[S(n-T_{a})x_{2}]+E[x_{1}x_{2}]E[S(n-T_{a})x_{1}]}{E[x_{2}^{2}]E[S(n-T_{a})x_{1}]-E[x_{1}x_{2}]E[S(n-T_{a})x_{2}]},$$ (8)

and

$$b=\frac{E[S(n)S(n-T_{a})]}{E[S^{2}(n-T_{a})]}.$$ (9)

After the extraction of the scaled estimate of the arterial signal (i.e., ${\mathrm{AC}}_{\mathrm{IR}}$) in $x_{1}$, the reference noise is extracted by removing the estimate of the arterial signal from $x_{1}$. This is quantified by the variance of the reference signal $N_{1}(n)$.

The mathematical expression of the reference signal $N_{1}(n)$ is

$$N_{1}(n)=x_{1}-\alpha S(n)=x_{1}-\alpha (x_{1}-cx_{2}).$$ (10)

By taking the gradient of $E[N_{1}^{2}(n)]$ with respect to α, the update rule for α is derived

$$\alpha =\frac{E[x_{1}^{2}]-cE[x_{1}x_{2}]}{E[S^{2}(n)]}.$$ (11)

In practice, $N_{1}(n)$ serves as the noise reference signal. Therefore, Eq. (10) is revised as

$$\frac{1}{1-\alpha }N_{1}(n)=x_{1}-\frac{\alpha c}{\alpha -1}x_{2}.$$ (12)

By comparing Eqs. (5) and (10), the update rule for parameter β is obtained

$$\beta =\frac{\alpha c}{\alpha -1}.$$ (13)

Thus, the linear combination of signals with parameter β yields an estimate of the noise reference signal N(n).

Once β is derived using the BSS technique, the two signals are combined in a weighted subtraction using β to produce the noise reference signal N(n) for the MSLMS adaptive filter. The fundamental period is applied in the FIR filter to generate the error signal $e_{1}(n)$, and the optimum β is updated using the update rules Eqs. (8) and (11) for α and c, respectively. This process is done on a frame basis, and for each frame, a new β is extracted, thereby yielding the most accurate noise reference signal through linear combination.

### 3.3 Entropy-Criterion-Based Frame-Selective MSLMS Adaptive Filter

Adaptive filtering algorithms modulate filter coefficients based on various adaptation strategies to suppress noise components that overlap in frequency with the desired signal. As shown in Fig. 7(c), the system requires both a reference signal and a desired signal. The reference signal is adaptively filtered and subtracted from the desired signal to produce a clean output.^33^ The LMS adaptive filter dynamically adjusts its weights using instantaneous gradient estimates to minimize output error. Its simple update rule and low computational cost make it a core algorithm in real-time signal processing. The standard LMS algorithm can be described as follows:

$$\mathrm{LMS}\text{ algorithm Filter output}:\;y(n)=W^{T}(n)N(n),$$ (14)

$$\text{Error signal}:\;e(n)=x_{2}(n)-y(n),$$ (15)

Weight update:  W(n+1)=W(n)+mue(n)N(n), (16)

where y(n) denotes the filter output, W is the weight vector, N(n) is the reference input, e(n) is the error signal, $x_{2}(n)$ is the desired signal (infrared PPG), and mu is the learning rate.

The algorithm is widely adopted owing to its computational simplicity and ease of implementation. However, its framework, characterized by fixed parameters (learning rate and filter order), presents an inherent contradiction in processing dynamic PPG signals: a smaller learning rate ensures convergence precision during stationary states but drastically reduces the tracking speed for sudden motion artifacts; conversely, a larger learning rate enhances tracking capability at the cost of introducing significant weight fluctuations and steady-state error during low-interference periods. Simultaneously, a fixed filter order is inadequate to accommodate motion artifacts of varying intensities and bandwidths. More critically, conventional LMS filters typically require a relatively stable initial phase (the “static convergence period”) for weight convergence—an assumption that often fails in the random and dynamic scenarios of real-world wearable monitoring, thus severely limiting their practicality.

To overcome these limitations, we propose a frame-selective MSLMS adaptive filtering mechanism. This mechanism constitutes an intelligent adaptive system with real-time state awareness and strategic switching capabilities. By establishing an external intelligent decision-making layer, it transforms the traditional adaptive filtering paradigm, which relies solely on internal weight iteration for convergence. The system first performs frame-based power spectral entropy analysis on the preprocessed IR PPG signal. The calculation equation is as follows:

$$H_{i}=-\underset{i}{\sum }(\frac{P_{xx}(i)}{\sum P_{xx}})\log_{2}(\frac{P_{xx}(i)}{\sum P_{xx}}+\varepsilon ),$$ (17)

where $P_{xx}(i)$ represents the normalized power spectral density of the i’th frequency bin, with subscript xx referring to the infrared signal’s power spectrum. A small constant ε is added for numerical stability. If the entropy value of a frame exceeds the threshold $H_{T}$, the frame is classified as a strong motion artifact interference frame (abbreviated as strong interference frame); otherwise, it is classified as a weak motion artifact interference frame (abbreviated as weak interference frame). To distinguish between weak interference frames and strong interference frames, power spectral entropy analysis is performed on PPG signals under different motion conditions. When the motion is smooth and artifact interference is weak, the entropy value is typically no more than 2; when the motion is intense and artifact interference is severe, the entropy value is typically higher than 2. Empirically, the threshold is set to $H_{T}=2$, which provides a sufficient margin between weak and strong interference.

During the system initialization phase, the adaptive filter is configured with baseline parameters: learning rate mu=0.0005 and filter order N=132. This configuration is solely employed to ensure transient stability for startup prior to obtaining the entropy-based decision for the first frame. Once the entropy value of the first signal frame is computed, the system immediately transitions into the intelligent adaptation process driven by real-time perception.

Based on the real-time entropy decision $H_{i}$, the intelligent system performs a strategic-level switch between two pre-optimized sets of filter parameters tailored for different scenarios, establishing a dual-mode processing scheme of “soft denoising” and “hard denoising” as illustrated in Fig. 7(d).

When $H_{i}\leq H_{T}$, the system determines that the current frame is subject to minimal motion artifact interference and enters the first-level soft denoising mode. This mode employs a smaller learning rate mu∈[0.0003,0.0007] and a lower filter order N∈[25,50]. The small learning rate ensures smooth weight updates, aiming to meticulously filter out background noise while maximizing the preservation of physiological details in the PPG waveform, thereby achieving high fidelity and low steady-state error.

When $H_{i}>H_{T}$, the system immediately detects high-intensity motion interference and executes a strategy switch, entering the second-level hard denoising mode. Here, the learning rate is expanded to mu∈[0.0008,0.0015], and the filter order is increased to N∈[51,120]. The key aspect of this design lies in the fact that the significantly increased learning rate *mu* intrinsically endows the algorithm with rapid convergence and dynamic tracking capabilities. By invoking the preset hard denoising mode, our system enables the filter to converge rapidly within an extremely short period following a motion state change, thereby re-establishing an effective noise suppression model. The increased order N provides the necessary degrees of freedom for modeling more complex, broader-band dynamic motion artifacts. Consequently, through its “perceive state – switch to dedicated strategy” approach, this system completely eliminates the reliance on a dedicated static initialization period for slow coefficient convergence.

To achieve locally optimal filtering under the current mode within each frame, the system employs a grid search algorithm to find the optimal parameter combination (mu*,N*) with the optimization objective of minimizing the error signal entropy

$$(mu*,N*)=\underset{\left(mu,N\right)}{\arg\;\min}\;H_{e(n)}(mu,N).$$ (18)

Therefore, the proposed frame-selective MSLMS adaptive filter, through the intelligent closed loop of “entropy assessment – mode selection – parameter optimization,” accomplishes a fundamental paradigm shift. By presetting parameter sets optimized for high precision and rapid convergence for stationary and motion states, respectively, the algorithm can dynamically and adaptively deliver efficient and robust motion artifact suppression across various scenarios, from any initial condition and in response to abrupt state transitions, thus providing a reliable guarantee for accurate heart rate estimation in dynamic environments.

## 4 Experimental Setup

**Fig. 8:**
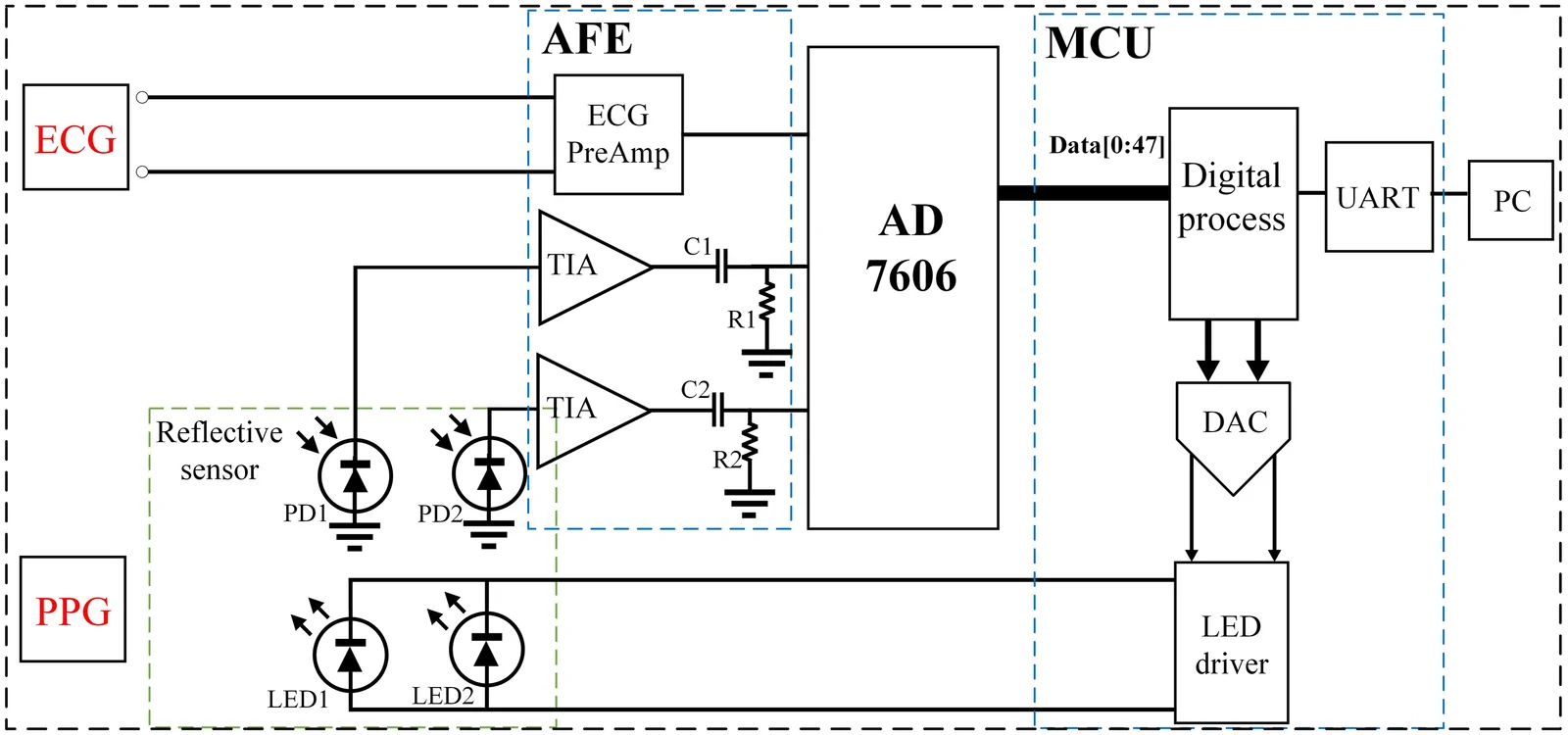
Real-time data acquisition system.

**Fig. 9:**
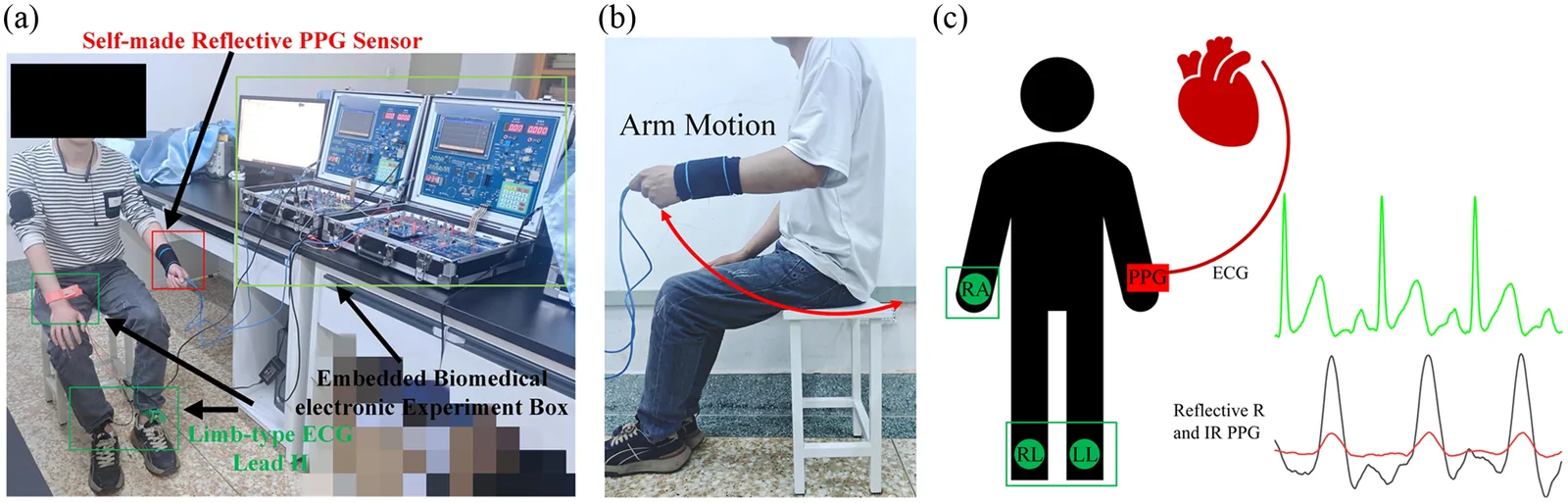
(a) Experimental data collection setup. (b) Arm performing a gait-like swinging motion with arbitrary amplitude and frequency. (c) Blood flow pathway from the heart to the limbs. The placement of sensors is shown on the left, whereas the expected waveforms are displayed on the right. RA, right arm electrode; RL, right leg; LL, left leg.

For the performance evaluation, a real-time data acquisition system, shown in Fig. 8, was used. The experimental acquisition setup is shown in Fig. 9(a), which simultaneously records lead II limb electrocardiogram (ECG) and wrist PPG data. As ECG is robust against motion artifacts, it was used as the reference for heart rate. PPG signals were acquired using the novel self-developed, multi-wavelength synchronous acquisition sensor based on a color filter array presented in this work.

To effectively validate the core mechanism of the algorithm while ensuring stable reference limb ECG signal quality, a controlled design was adopted: subjects remained seated while performing arbitrary amplitude and frequency swings with the left arm only, shown in Fig. 9(b). This paradigm is a classic method for inducing motion artifacts, effectively simulating interference introduced during daily activities whose spectrum may overlap with the PPG signal, and has been widely used for preliminary algorithm validation.

The specific experimental protocol, shown in Fig. 9(c), consists of a 10-s stationary period to collect PPG signals unaffected by motion artifacts, followed by a 16-s period of left-arm swinging with arbitrary amplitude and frequency to introduce motion artifacts. This cycle was repeated three times to complete the controlled experimental session. The sensor was secured with an elastic wrist strap to ensure firm skin contact, simulating an ideal wearing condition.

The experiment was approved by the Medical Ethics Committee of the School of Medicine at Kunming University (Approval No. 2023007). All participants provided written informed consent prior to data collection and submitted basic demographic information, including gender, age, height, and weight. A total of 13 healthy subjects aged 18 to 27 years were recruited. All collected data were encrypted and stored in a secure database to protect participant privacy.

This controlled experiment was designed to establish a foundation for verifying the core principle of the BSS-MSLMS algorithm in exploiting the correlation of motion artifacts across multiple wavelengths, with future work extending to more complex motion scenarios.

## 5 Experimental Removal and Analysis

### 5.1 Performance of the Proposed Motion Artifact Removal Algorithm

To ensure amplitude consistency of the PPG signals and facilitate computation by the proposed algorithm, the signals were first normalized to the range of −1 to 1. Subsequently, a preprocessing step was applied to suppress high-frequency noise, smooth the waveform, and stabilize the PPG baseline, thereby improving the overall signal quality. Given that the AC components of PPG signals typically fall within the frequency range of 0.5 to 5 Hz, a fourth-order Butterworth bandpass filter was employed for signal conditioning. The signals were sampled at 1000 Hz, with a low cutoff frequency of 0.5 Hz and a high cutoff frequency of 5 Hz.^34^

**Fig. 10:**
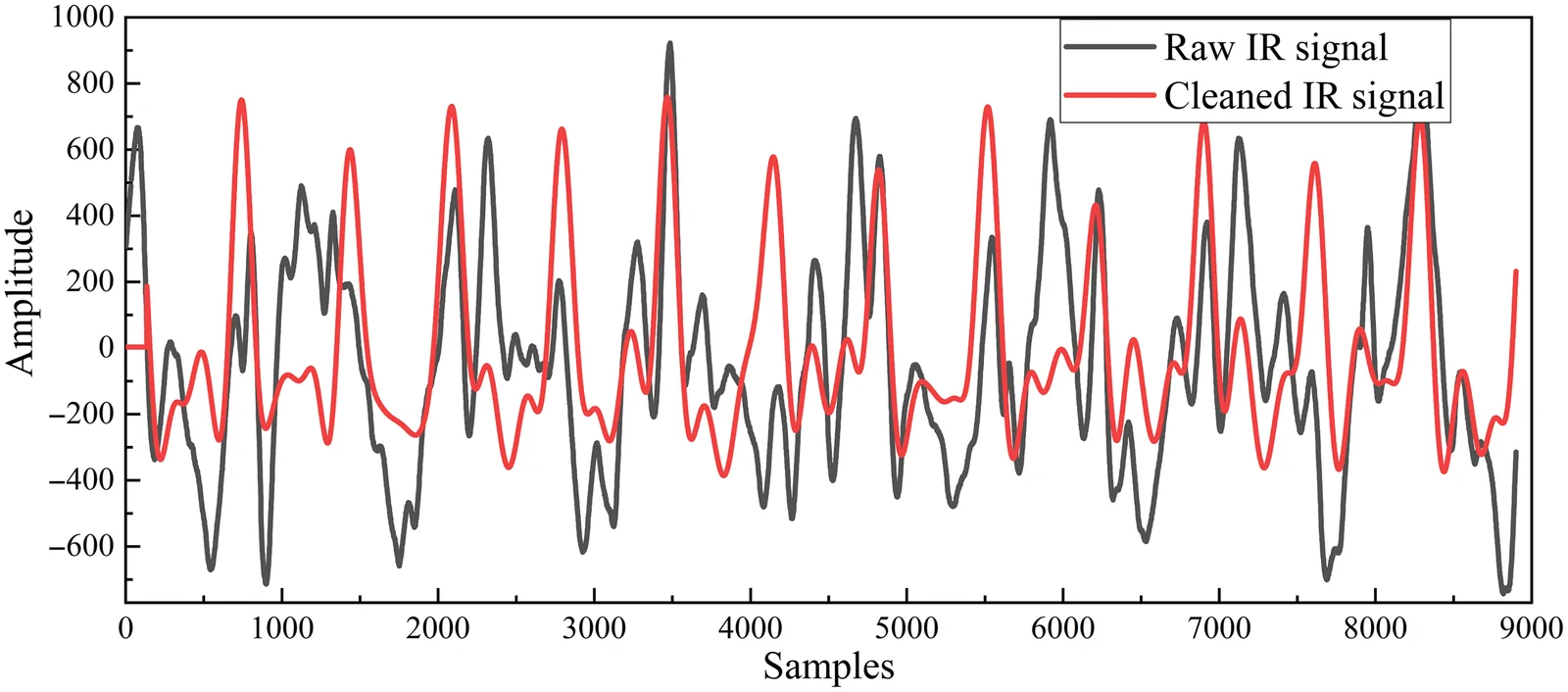
Comparison of raw PPG waveform and cleaned signal using BSS-MSLMS adaptive filtering for MA removal.

To validate the effectiveness of the proposed motion artifact removal algorithm, Fig. 10 presents a comparison between the raw PPG signals and the clean PPG signals obtained after applying the proposed motion artifact removal algorithm. As illustrated, the raw PPG signals are significantly distorted in both waveform shape and periodicity due to motion interference. In contrast, the PPG signals reconstructed by the proposed algorithm demonstrate clear periodicity and successfully recover key morphological features, thereby enhancing their reliability for subsequent analysis and interpretation. It is worth emphasizing that although the motion in this experiment was of relatively moderate amplitude and the sensor maintained firm contact, the spectrum of the artifacts introduced by such motion highly overlaps with the physiological pulse wave band. This creates a typical “spectral aliasing” scenario where traditional frequency-selective filtering methods often fail. The algorithm’s success in reconstructing a clean waveform with clinical reference value under this challenging scenario precisely demonstrates the efficacy of its core approach: leveraging the correlation of motion artifacts from a common origin across multi-wavelength signals within the BSS-MSLMS adaptive framework for source separation. This also provides a proof of principle for the algorithm’s applicability under more complex motion conditions.

**Fig. 11:**
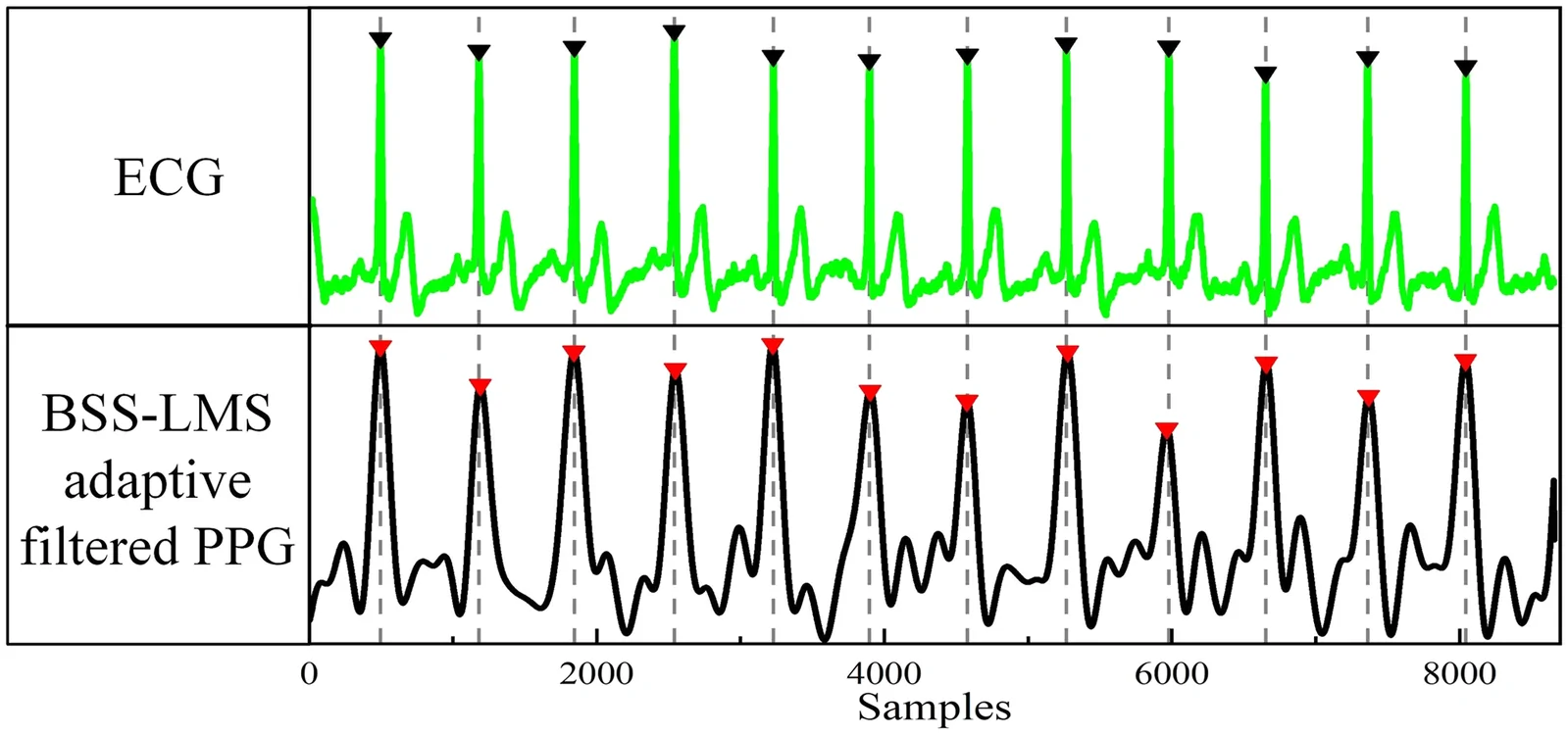
Comparison of peak positions between synchronously measured limb-lead ECG and cleaned PPG signal processed by the proposed algorithm.

To verify whether the cleaned PPG signal retains HR information, its peak locations were compared with the R-peaks of the simultaneously measured limb lead II ECG in Fig. 11. For synchronization of the ECG and cleaned PPG signals, the cross-correlation between the peak positions in both ECG and PPG signals is calculated. The delay among the signals corresponds to the time interval that results in the highest correlation value. This delay is then used to adjust the signals ensuring their synchronization. As shown in the figure, the peak positions of the cleaned PPG signals, after motion artifact removal using the proposed algorithm, are well aligned with the R-peaks of the ECG signals. This demonstrates that the proposed algorithm effectively preserves critical HR information even in dynamic motion scenarios. Owing to its motion artifact suppression capability and dynamic parameter optimization mechanism, the algorithm enhances the quality and stability of PPG signals in complex environments. This contributes to mitigating the inaccuracies in traditional PPG measurements caused by motion interference, thereby offering a viable solution for vital sign monitoring in dynamic scenarios.

### 5.2 Heart Rate Monitoring

To evaluate the performance of the proposed algorithm in heart rate monitoring, three metrics for HR were calculated: Pearson correlation coefficient, mean absolute error, and Bland–Altman analysis.

Pearson correlation coefficient is defined as

$$R=\frac{\mathrm{Cov}({\mathrm{HR}}_{\mathrm{est}}.{\mathrm{HR}}_{\mathrm{ref}})}{\sigma {\mathrm{HR}}_{\mathrm{est}}.\sigma {\mathrm{HR}}_{\mathrm{ref}}},$$ (19)

where $\mathrm{Cov}({\mathrm{HR}}_{\text{est}}.{\mathrm{HR}}_{\text{ref}})$ denotes the covariance between the estimated heart rate and the reference heart rate, and σ represents the standard deviation.

Mean absolute error (MAE) is calculated as

$$\mathrm{MAE}=\frac{1}{N}\overset{N}{\underset{i}{\sum }}|{\mathrm{HR}}_{\mathrm{est}}(i)-{\mathrm{HR}}_{\mathrm{ref}}(i)|,$$ (20)

where N is the length of the HR sequence, ${\mathrm{HR}}_{\mathrm{est}}(i)$ is the HR estimated at time i using the BSS-MSLMS adaptive filtering algorithm, and ${\mathrm{HR}}_{\mathrm{ref}}(i)$ is the reference HR at time i.

Bland–Altman plot: In this plot, the x-axis represents the mean of the estimated and reference HR values, whereas the y-axis shows their difference. The limit of agreement (LOA), which is defined as [μ−1.96σ,μ+1.96σ], where μ is the average difference and σ is the standard deviation.

HR values estimated from PPG signals were compared with HR reference values derived from ECG. HR was extracted every 8 s, resulting in 104 pairs of HR measurements. Table 3 shows that heart rate estimates obtained via the proposed BSS-MSLMS adaptive filtering algorithm demonstrated significant improvements across all metrics relative to estimates derived from unfiltered raw PPG signals. Specifically, the LOA between the raw PPG signal and ECG-derived HR was [3.09, 26.94] bpm, indicating substantial deviation and a high risk of HR misestimation. In contrast, application of the proposed algorithm narrowed the LOA significantly to [−1.98,2.11]  bpm, greatly enhancing HR estimation accuracy and reducing the likelihood of misclassification or missed detections. In addition, the Pearson correlation coefficient between PPG- and ECG-derived HR improved to 0.99 after artifact removal, reflecting a 97.23% relative increase and indicating strong consistency in HR trend capture. More importantly, the MAE was reduced to 0.75 bpm—a 94.94% relative reduction—bringing HR estimation within clinically acceptable error margins and substantially improving the reliability of vital sign monitoring.

**Table 3:** Performance comparison of the HR evaluation between the cleaned PPG signal obtained via the proposed BSS-MSLMS adaptive filtering algorithm and the raw PPG signal.

Parameters	R	MAE (bpm)	LOA (bpm)
Raw and ECG HR	0.8691	15.1292	[3.09, 26.94]
BSS-MSLMS and ECG HR	0.9964	0.7651	[−1.98, 2.11]

The comprehensive improvement in these performance metrics not only validates the effectiveness of the proposed algorithm in separating motion artifacts from physiological signals under spectrally overlapping conditions in a controlled experiment but also provides a solid foundation for its potential application in real-world daily activities. Motion interference in daily activities often features varying amplitudes and frequencies that interweave with the heart rate band. The mechanisms our algorithm relies on—modeling the common-origin motion correlation across multiple wavelengths and the frame-selective adaptive filtering—constitute an effective solution precisely targeting such complex spectral aliasing problems.

**Fig. 12:**
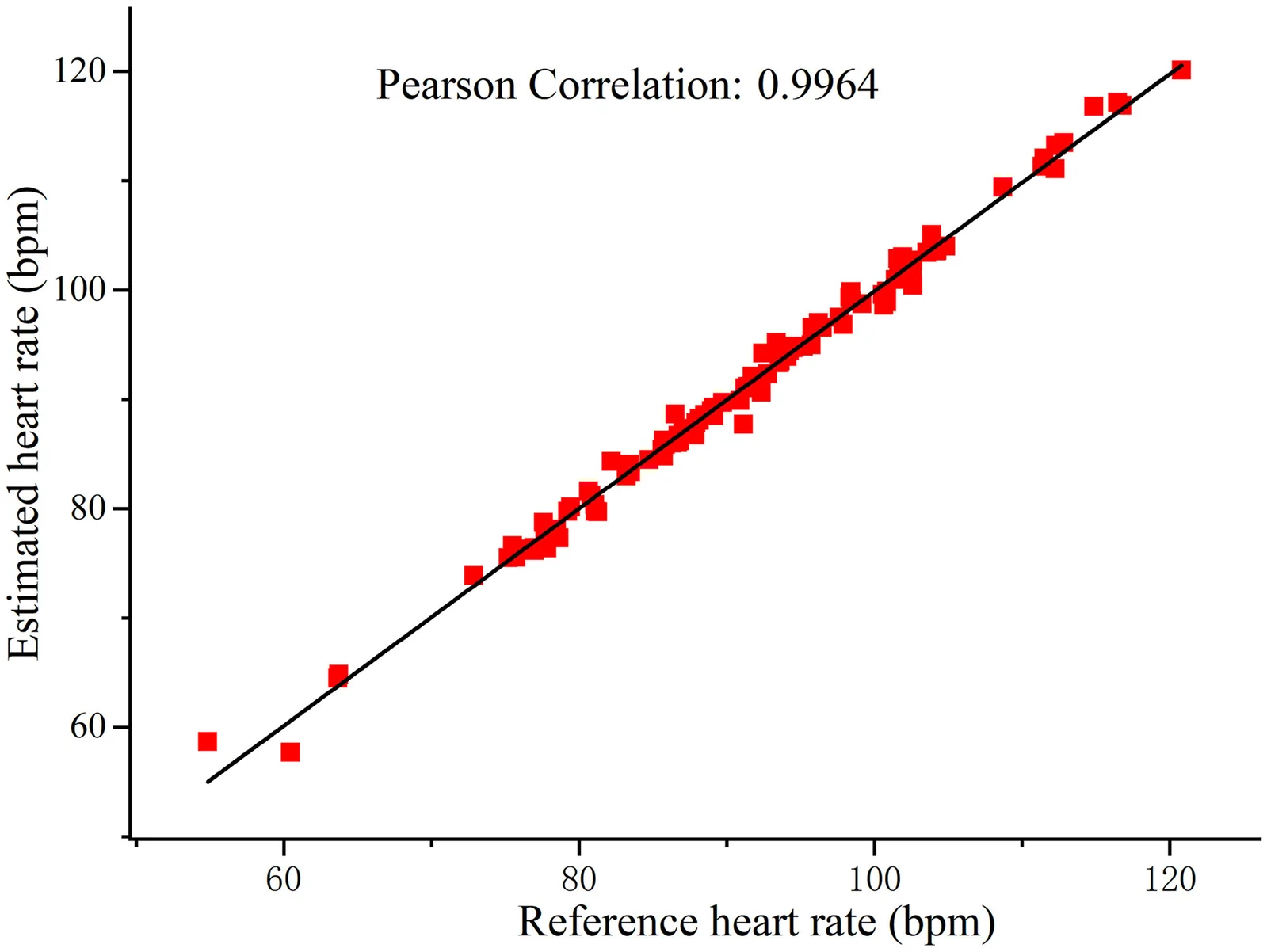
Pearson correlation plot between HR estimated by the proposed algorithm and the reference HR.

To evaluate the accuracy of HR estimates from the proposed algorithm, we generated both a Pearson correlation plot and a Bland–Altman plot. The Pearson correlation plot visually illustrates the linear relationship between the estimated HR values and the reference HR values, reflecting the algorithm’s precision and consistency in heart rate detection. As shown in Fig. 12, a strong linear relationship is observed between the reference HR and the HR estimated from the motion-artifact-free PPG signal processed by the BSS-MSLMS adaptive filtering algorithm. The Pearson correlation coefficient is nearly 1, indicating a high degree of agreement with the clinical gold standard ECG measurement.

**Fig. 13:**
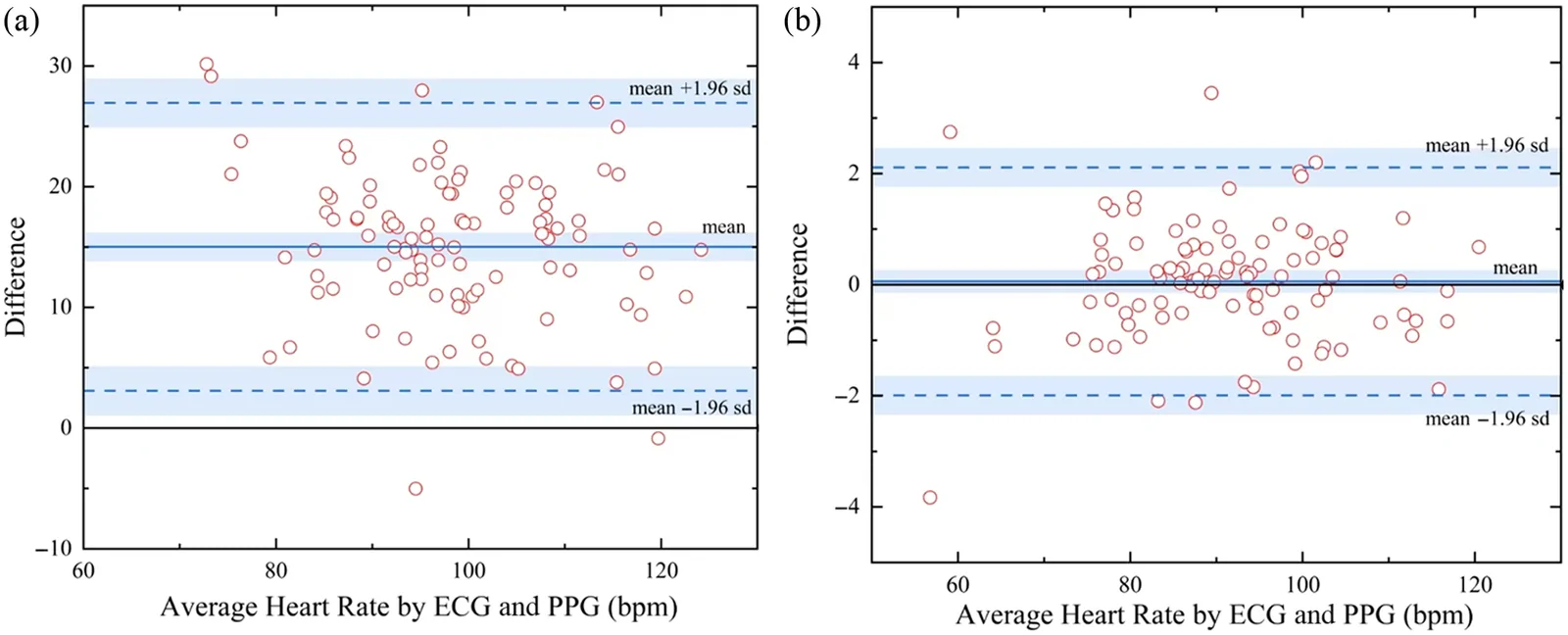
(a) Bland–Altman plot of HR estimates from raw PPG signal versus ECG reference before MA removal. (b) Bland–Altman plot of HR estimates from cleaned PPG signal versus ECG reference after MA removal.

The Bland–Altman plot was used to analyze the agreement between the estimated HR and the reference HR. Figures 13(a) and 13(b) respectively show the Bland–Altman plots before and after motion artifact removal. As seen in the figures, the HR estimates from the raw, noise-contaminated PPG signal show substantial deviations from the reference HR, which could lead to misjudgments or missed detections in clinical monitoring. After applying the proposed algorithm, the agreement between estimated and reference HR values improved markedly. The data points cluster more closely around the mean difference line, significantly enhancing the reliability of PPG-based HR monitoring in both daily activity.

### 5.3 Comparative Analysis

The proposed motion artifact removal algorithm was compared with some state-of-the-art HR estimation methods reported in Refs. 15, 22, 24, and 35 in Table III, unlike the methods in Refs. 15 and 35. In contrast to the complex signal processing approaches used in Refs. 15 and 35—such as triaxial accelerometer decomposition, variational mode decomposition (VMD), and the combination of multiple RLS and NLMS adaptive filters—the proposed algorithm does not rely on external motion sensors, resulting in a more streamlined system design. This significantly reduces device size, enhancing wearability and practicality in clinical settings.

Regarding performance metrics, as shown in Table 4, there are notable differences in the motion scenarios defined between this study and the referenced works. Studies^15^^,^^35^ primarily evaluated activities such as walking and running, whereas Refs. 22 and 24 involved more complex and intense motions such as multi-directional finger movements or full-body activities. In contrast, this study focuses on free-arm-swinging during gait—a typical high-frequency, low-amplitude activity that is more representative of the most common and practical application scenario for wrist-worn devices. Despite this relatively singular yet more universal motion condition, the MAE achieved by our algorithm is 0.76 bpm, which remains significantly lower than the results reported in the literature under various motion patterns, demonstrating superior estimation accuracy. Although the Pearson correlation coefficient (0.99) reported in Ref. 15 is marginally comparable to ours (0.99), our algorithm achieves a substantially narrower limit of agreement ([−1.98,2.11]  bpm), indicating higher consistency with the ground truth and more stable, reliable motion artifact suppression. This outcome validates, on one hand, the high correlation of MA components induced by common-origin motion within strictly synchronized multi-wavelength PPG signals, providing a foundation for subsequent separation. On the other hand, it demonstrates that the two-stage adaptive filtering strategy adopted in this work is structurally superior to the single-type adaptive filters used in Refs. 22, 24, and 35, enabling more effective extraction and suppression of common-mode motion interference and thereby achieving better overall performance.

**Table 4:** Comparison with motion artifact removal techniques for heart rate estimation using multi-wavelength PPG signals.

	Ref. 22	Ref. 24	Ref. 15	Ref. 35	This work
Method	NLMS	SS-LMS	V-DERMANC^b^	NLMS + RLS	BSS-MSLMS
Measurement site	Finger	Finger	Wrist	Wrist	Wrist
No. of PPG channels	2	2	2	2	2
Window size (s)	6	2 to 3	8	8	8
Light wavelengths of LEDs	Red (660 nm)	Red (630 nm)	Orange–red (609 nm)^c^	—	Red (660 nm)
	IR (895 nm)	Green (523 nm)			IR (940 nm)
Motion scenarios	Hand motions^a^ walking	Walking	Walking	Arm motions^d^	Arm motions^e^
	Running	Running	Running	Running	
	Standing	Squatting			
No. of subjects	6	6	12	23	13
${\mathrm{MAE}}_{\mathrm{HR}}$ (bpm)	—	1.1/1.8/1.5	0.95	1.03	0.76
Corr	0.98	—	0.997	0.9895	0.99
LOA	[−7.0, 5.9]	—	[−3.26, 3.04]	[−8.6, 8.4]	[−1.98, 2.11]

a Vertical/horizontal finger movements and finger flexion.

b Uses two modes from each of the three accelerometer channels as reference signals for MANC (LMS-based), with modes extracted using VMD.

c Two orange–red LEDs with a wavelength of 609 nm.

d Arm-involved activities such as push-ups, boxing, and swimming.

e Arm swinging in a gait-like motion with arbitrary amplitude and frequency.

When further compared with studies,^22^^,^^24^ which also perform MA removal based on multi-wavelength PPG signals, the performance advantage of our algorithm primarily stems from fundamental improvements in the hardware architecture. The fingertip-clip sensors used in Refs. 22 and 24, constrained by single-point measurement and time-division multiplexing driving mechanisms, struggle to achieve strictly synchronized acquisition of multi-wavelength signals. This leads to reduced correlation of MA components across different channels due to temporal misalignment and differences in motion coupling.

In contrast, our self-developed wrist-worn sensor, utilizing synchronized multi-wavelength LED driving and an integrated color filter array, enables strictly simultaneous acquisition of multi-channel PPG signals. Wrist-measured PPG signals typically have a smaller AC component compared with finger PPG, posing different requirements for signal processing and further underscoring the critical importance of hardware synchronization. This co-designed hardware ensures that MA components induced by the same motion source maintain high temporal synchrony and morphological consistency across different wavelength signals. Consequently, it provides a strongly correlated data foundation for the BSS-MSLMS algorithm to establish a high-precision cross-wavelength coupling model. Therefore, although the core algorithmic concept in all cases exploits MA correlation, the breakthrough in hardware-synchronized acquisition in this work fundamentally enhances signal quality, which is the key reason for the algorithm’s significantly superior performance compared with Refs. 22 and 24.

## 6 Conclusion

This paper proposes a frame-selective BSS-MSLMS adaptive filtering algorithm for motion artifact removal in multi-wavelength PPG signals. The algorithm enables accurate heart rate extraction from wearable PPG sensors. The proposed technique leverages the PPG sensor’s synchronized multi-wavelength acquisition capability to model physiological PPG signals and motion artifacts, exploiting the high correlation of MA components across wavelengths due to common motion interference. A highly correlated noise reference is dynamically generated via BSS. Coupled with a power spectral entropy-based signal quality assessment mechanism, this enables adaptive switching and optimization of filter parameters. Consequently, the method effectively overcomes the dependency on a static convergence period typical of conventional methods, significantly enhancing the tracking and suppression capability for dynamic motion artifacts. In controlled experiments simulating daily activities involving 13 subjects, the method demonstrated excellent performance. The processed HR estimates achieved a MAE of 0.76 bpm, LOA of [−1.98,2.11]  bpm, and a Pearson correlation coefficient of 0.99, indicating a substantial improvement in the accuracy of dynamic heart rate monitoring.

The current study primarily validated the algorithm under laboratory conditions for the typical daily scenario of mild arm swinging. Its generalizability to more intense or complex motion patterns requires further exploration. Furthermore, the experiments employed near-ideal sensor-skin contact conditions to ensure signal stability, which differs from real-world wearing scenarios where contact may vary due to strap tightness, perspiration, etc. The robustness of the algorithm needs thorough evaluation under conditions closer to practical use.

Future work will focus on integrating the algorithm into a more compact wearable prototype. Large-scale testing in real-world dynamic scenarios such as outdoor walking and exercise, along with comparisons against mainstream commercial devices, will be conducted to comprehensively evaluate its practical performance. Simultaneously, by further capitalizing on the hardware advantage of synchronous multi-wavelength acquisition, we will explore extending the algorithmic framework to the non-invasive, motion-robust measurement of additional physiological parameters, such as blood oxygen saturation. This aims to advance wearable health monitoring technology toward higher clinical standards and broader application scenarios.

## Data Availability

The photoplethysmogram data supporting the findings of this study were collected using a self-developed sensor by the authors. The raw data are not publicly available to protect participant privacy and in accordance with the terms of the informed consent agreements signed by the participants. These data, which contain personal physiological information, are restricted for use in this study and its directly related follow-up analyses. Controlled access for research purposes may be requested from the corresponding author at zoumei@kmu.edu.cn.
