# Color-filter-array-based multispectral photoplethysmography optical sensor and its motion artifact correction algorithm (Erratum)

**DOI:** 10.1117/1.JBO.31.6.069801

**Published:** 2026-06-10

**Authors:** Yunfen Wei, Shu Cong, Runchao Yan, Zekai He, Hong Li, Gongming Yu, Mei Zou

**Affiliations:** a Kunming University, School of Physical Science and Technology, Kunming, China; b Nanjing University, National Laboratory of Solid State Microstructures, Nanjing, China

## Abstract

The erratum lists corrections to the published article.

This article [*J. Biomed. Opt.* **31**(3), 037001 (2026) [DOI: 10.1117/1.JBO.31.3.037001] was originally published on 16 March 2026 with the following errors:

• In the x axis of Figure 6, the labels were swapped, with “Motion state (MA)” on the left and “Resting state (AC)” on the right; the corrected figure (below) includes the same labels but with “Resting state (AC)” on the left and “Motion state (MA)” on the right, as follows:
• On page 11, there were two errors.
  ○ The final sentence of the first paragraph stated, “Frames with entropy exceeding a threshold HT are classified as noise contaminated, otherwise as clean.”
    ▪ This description is incorrect. The corrected description is as follows:
      • If the entropy value of a frame exceeds the threshold HT, the frame is classified as a strong motion artifact interference frame (abbreviated as strong interference frame); otherwise, it is classified as a weak motion artifact interference frame (abbreviated as weak interference frame).
  ○ The second paragraph stated, “To distinguish noisy from clean frames, power spectral entropy is analyzed for PPG signals under motion and stationary conditions. During stationary periods, entropy values are low and stable across frames; in contrast, motion results in higher and more fluctuating entropy values. Empirically, a threshold of $H_{T}=2$ provides a sufficient margin for separating the two states.”
    ▪ This description is incorrect. The correct description is as follows:
      • To distinguish between weak interference frames and strong interference frames, power spectral entropy analysis is performed on PPG signals under different motion conditions. When the motion is smooth and artifact interference is weak, the entropy value is typically no more than 2; when the motion is intense and artifact interference is severe, the entropy value is typically higher than 2. Empirically, the threshold is set to $H_{T}=2$, which provides a sufficient margin between weak and strong interference.

The article was corrected and republished 28 May 2026.

These corrections do not affect the core conclusions or scientific value of the article.

